# New insights on potency assays from recent advances and discoveries in CAR T-cell therapy

**DOI:** 10.3389/fimmu.2025.1597888

**Published:** 2025-05-08

**Authors:** Lipei Shao, Yanyan Zheng, Robert P. Somerville, David F. Stroncek, Ping Jin

**Affiliations:** Center for Cellular Engineering, Clinical Center, National Institutes of Health, Bethesda, MD, United States

**Keywords:** potency assay development, CAR T cell therapy, multi-omics, advances and discoveries, quality control

## Abstract

This review explores recent advances in the characteristics and manufacturing of CAR T-cell products. Traditional potency assays have been designed based on well-established CAR T-cell functionalities. However, the advent of innovative tools and methodologies has revealed a broader spectrum of important CAR T-cell characteristics that correlate with function. Furthermore, as manufacturing strategies continue to evolve, conventional potency assays may no longer fully capture the complexity of these products. Therefore, it is essential to examine these emerging characteristics and manufacturing approaches and consider the development of tailored potency assays to ensure products are fully characterized.

## 1 Introduction

Chimeric antigen receptor (CAR) T-cells demonstrate promising clinical outcomes (1–7), as the indications for the use of these therapies are growing there is a need to develop appropriate and robust potency assays that can accurately assess their therapeutic potential. Potency assays are generally designed to measure the biological activities of CAR T-cells based on their mechanism of action (MoA) (8, 9). The MoA of CAR T-cells is a multifaceted process that underlies their therapeutic effects on target cells. CAR T-cells are designed to express chimeric antigen receptors that specifically recognize and bind to antigens on the surface of target cells (1–4, 10–14). Upon antigen recognition, CAR T-cells become activated, initiating a cascade of cellular responses that ultimately lead to the destruction of target cells (15). Beyond their immediate cytotoxic functions, their viability and *in vivo* expansion and persistence are critical for sustained therapeutic effect (15–17).

Understanding the key components of MoA is essential for developing potency assays that accurately reflect the functional capabilities of CAR T-cells and ideally, these will correlate with clinical outcomes. The potency of the FDA-approved CAR T-cell products is primarily assessed by measuring the release of IFN-γ in response to target cells, along with other factors, such as cell viability, and product-specific attributes, such as the expression of the specific target CAR (8, 9).

Over the past decade, advances in CAR T-cell research have led to the identification of new cellular characteristics associated with clinical responses and innovative manufacturing procedures have been developed to enhance these characteristics, driven by emerging tools and methodologies. These advances raise concerns that conventional potency assays may not fully capture the complexity of manufactured products. Given these developments, it is crucial to comprehensively review recent progress in the design and production of CAR T-cells and explore the need for tailored potency assays that fully define manufactured products.

## 2 Advances in CAR T-cell product profiling

**Figure 1:**
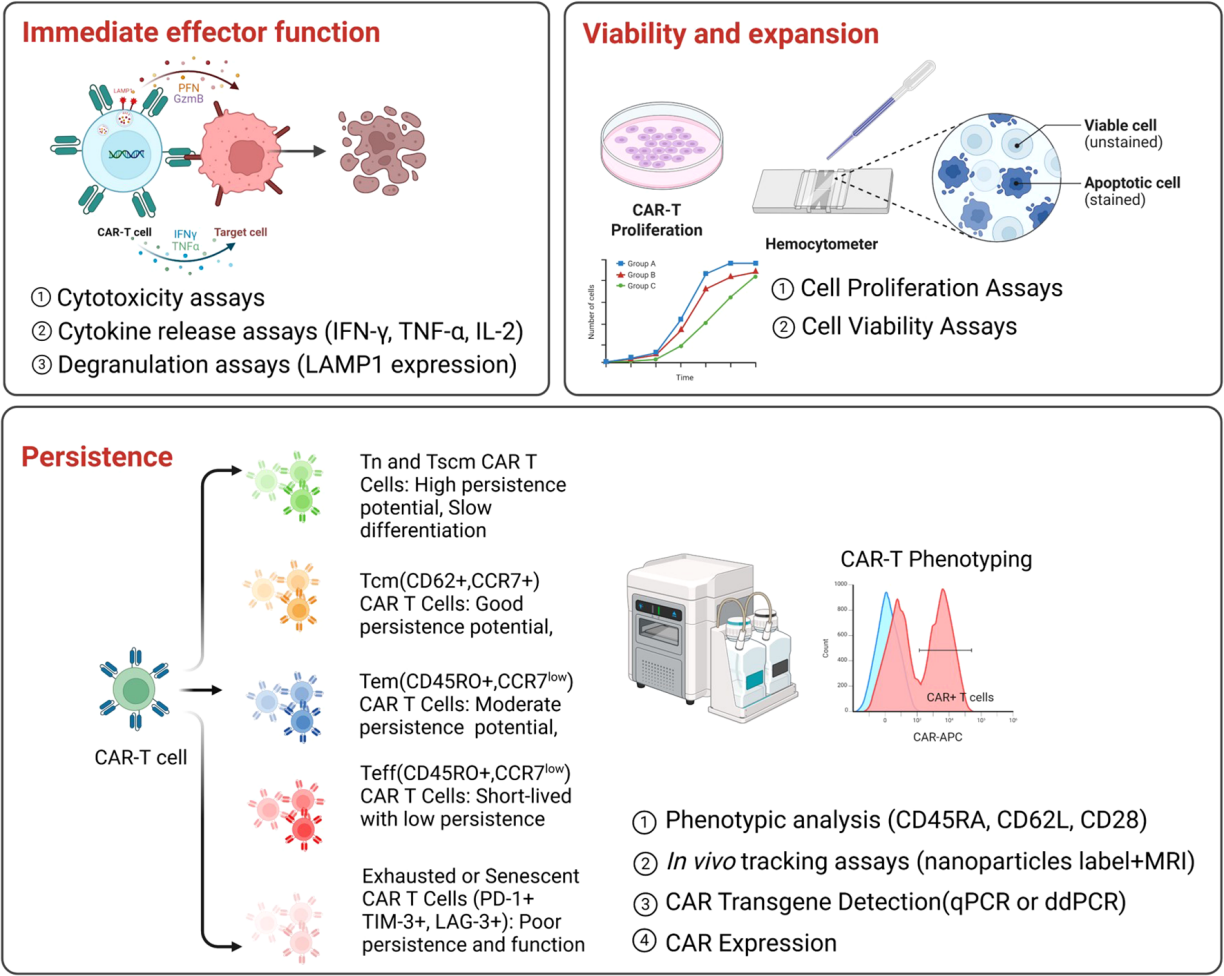
Current potency assays for CAR T-cell products. Key functional assays used to evaluate CAR T-cell potency, categorized into three main aspects.Upper panel (left): Evaluation of immediate effector function, by measuring cytotoxicity, cytokine release (e.g., IFN-g, TNF-a, IL-2), and degranulation (e.g., LAMP1 expression). Upper panel (right): Evaluation of viability and expansion by assessing cell proliferation and viability. Bottom panel: Evaluation of persistence by analyzing CAR T-cell phenotypes, performing in vivo tracking, and assessing CAR transgene expression at pre-infusion and post-infusion.

**Figure 2:**
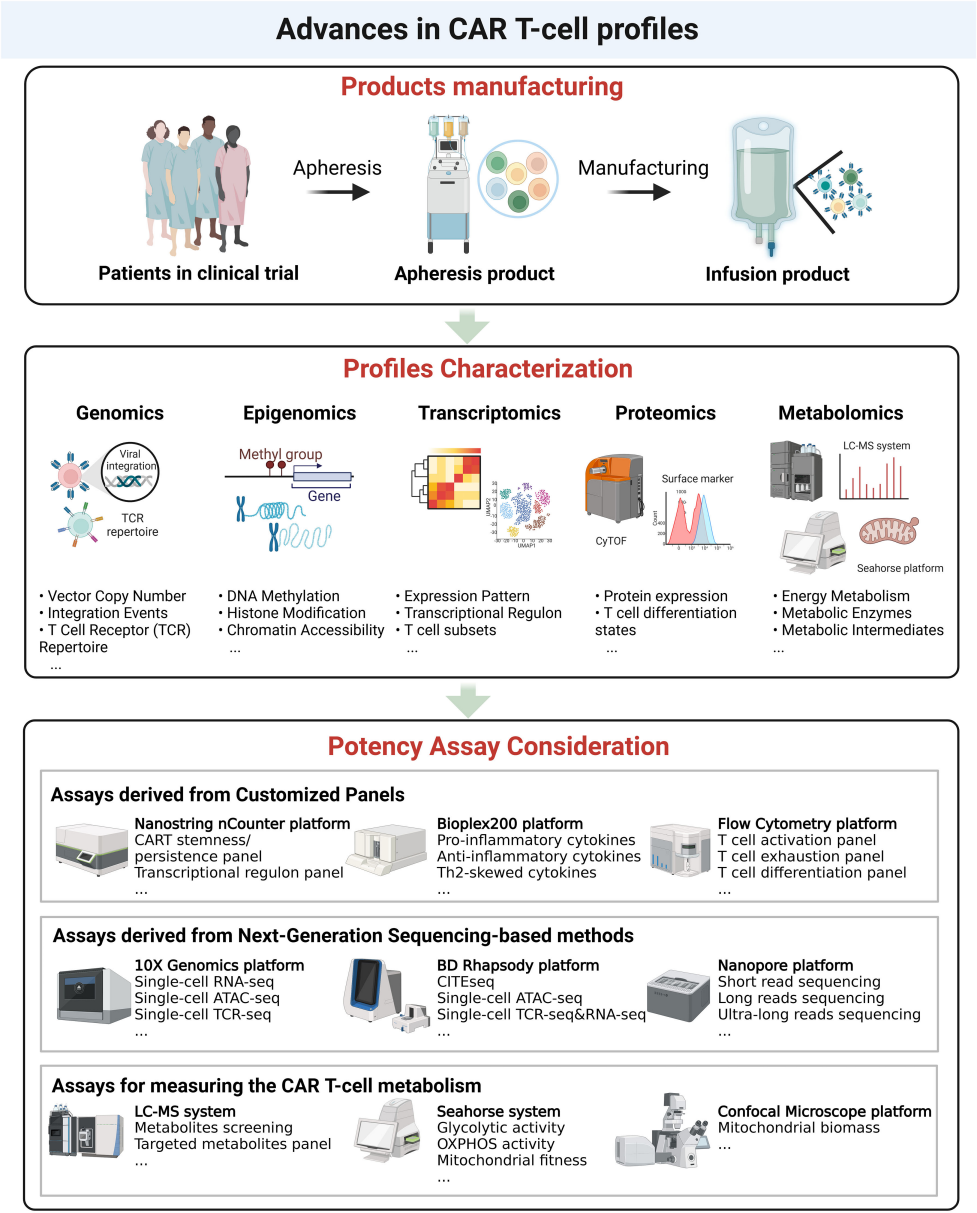
Advances in CAR T-cell analysis. Upper panel. Overview of CAR T-cell product manufacturing, from apheresis collection to infusion of the final CAR T-cell product. Middle panel. Characterization of CAR T-cell profiles at multiple molecular levels, including genomics (vector copy number, integration events, TCR repertoire), epigenomics (DNA methylation, histone modifications, chromatin accessibility), transcriptomics (expression patterns, transcriptional regulation, T-cell subsets), proteomics (protein expression, T-cell differentiation states), and metabolomics (energy metabolism, metabolic enzymes, intermediates). Bottom panel. Potency assay considerations, including customized panel-based assays (e.g., cytokine profiling, T-cell activation), next-generation sequencing-based approaches (e.g., single-cell RNA-seq, ATAC-seq, TCR-seq), and metabolic assessment methods (e.g., glycolytic activity, mitochondrial fitness).

Evaluating CAR T-cell potency requires a comprehensive matrix of assays that fully profile the key activities and characteristics of the cells (**Figure 1**). Over the past decade, advanced multi-omics approaches, including genomics, epigenomics, transcriptomics, proteomics, and metabolomics at both bulk and single-cell resolution, have significantly enhanced our understanding of CAR T-cell function at the molecular level (**Table 1**). In this section, we review these advancements and highlight key insights that may guide the development of next-generation potency assays (**Figure 2**).

**Table 1:** Representative multi-omics profiling approaches applied in the CAR T-cell products.

Type	Method	Purpose
Genomics	Stage DNA-seq	Vector integration sites detection (21, 36–40)
	Bulk TCR-seq	T-cell receptor repertoire/diversity/clonality (24, 25, 42–50)
	Single-cell DNA-seq	Check the vector integration sites at single-cell resolution (39)
Epigenomics	ATAC-seq	Characterization of chromatin accessibility across whole genome (94, 95)
	scATAC-seq	Characterization of chromatin accessibility across whole genome at single-cell level (70, 94)
	ChIP-seq	Identification of specific transcriptional factor binding sites across whole genome
	DNase-seq	Identification of regulatory regions of the genome
	Methyl-seq	Check the DNA methylation condition across whole genome (59, 62, 63)
Transcriptomics	Bulk RNA-seq	Analyzing the expression of transcripts across bulk cells (5, 75, 76, 78, 94, 95)
	Single-cell RNA-seq	Measuring the expression of transcripts in individual cells (22, 50, 78, 81, 82, 85, 94, 95, 101)
	Single-cell V(D)J RNA-seq	Simultaneously analyzing gene expression and TCR repertoire (24, 48–50)
Proteomics	CyTOF	Simultaneously measuring multiple protein markers (102, 108, 111)
	IMC/MIBI-TOF	Enable highly multiplexed spatial imaging of cells
	Mass Cytometry	Analyzing co-regulation and crosstalk between cellular programs (105, 106, 109)
Metabolomics	Seahorse XF Analyzer	Measuring real-time cellular metabolism (mitochondrial respiration and glycolysis) (75, 94, 95, 114, 116, 124)
	LC-MS/MS	Identification and quantification of metabolites, proteins, and lipids in the supernatant (110, 114)
	Met-Flow	Analyze single-cell metabolism by combining flow cytometry with metabolic probes

ATAC-seq, Assay for Transposase Accessible Chromatin using Sequencing.

CYTOF, Cytometry by Time-of-Flight.

MIBI-TOF, Multiplexed lon Beam Imaging by Time-of-Flight.

### 2.1 Genomic profiles in CAR T-cell products

In recent years, the genomic profiling of CAR T-cell products has primarily focused on vector copy number (VCN) (9, 18, 19), and vector integration sites (8, 20, 21). Additionally, immunogenomic analyses, such as bulk and single-cell T cell receptor (TCR) sequencing (TCR-seq), have been applied to assess the TCR repertoire in CAR T-cells (22–25). For FDA-approved CAR T-cell products, VCN quantification is a mandatory component of lot-release testing, with droplet digital PCR (ddPCR) being widely used as a routine safety assay to measure VCN in most quality control (QC) laboratories (8, 9). Here, we focus on recent advancements in vector integration and TCR repertoire profiling.

On November 28, 2023, the U.S. FDA announced an investigation into cases of secondary malignancies in patients who received CAR T-cell therapy (20). The potential risk for secondary malignancies caused by insertional mutagenesis has long been a concern in CAR T-cell therapy, as viral vector transduction is required for CAR expression. However, the exact nature and frequency of genotoxicity risk associated with retroviral or lentiviral insertion remains unclear and deserves thorough investigation and transparency. Recent large-cohort follow-up studies report the incidence of secondary malignancies after CAR infusion ranging from 2-16% (26–31). Insertional mutagenesis occurs when viral vector integrates into a gene associated with cancer development, inadvertently activating an oncogene or inactivating a tumor suppressor gene and potentially leading to oncogenesis. Beyond this risk, vector integration has, in certain circumstances, been associated with a selective growth advantage, resulting in clonal CAR T-cell expansion, dominance, and persistence (32–34).

Research by Carl June’s team revealed that the integration events at the *TET2* gene enhanced CAR T-cell potency (32). Similarly, an NCI research group identified clonal expansion of CAR T-cells harboring lentivector integration in the *CBL* gene following CAR T-cell therapy (33). Christopher et al. demonstrated that both the number and genomic loci of integration events correlate with clinical outcome in CD19 CAR T-cell products (35). Their study found that genes with integration sites enriched in responders were commonly involved in cell-signaling and chromatin modification pathways, suggesting that insertional mutagenesis in these genes promoted therapeutic T-cell proliferation. However, the consequences of viral vector integration into these reported genes have not been consistently reproducible (32). A study on the clonal dynamics of CAR T-cells over time found that not all T-cells with *TET2* integration exhibit expansion, either during CAR T-cell production or after infusion (34). These findings underscore the importance of monitoring vector integration sites with potency assays in order to address safety and efficacy concerns. Our team and other research groups have developed robust pipelines for detecting viral insertion events (21, 36–40). The Bushman lab initially applied the Illumina sequencing method to investigate viral integration events in cellular products and developed the INSPIIRED pipeline (36), which enables measurement of integration events at bulk-cell resolution. Furthermore, Wenliang Wang and colleagues developed the EpiVIA pipeline, which enables detection of integration sites at the single-cell level (39). These advancements have significantly improved the feasibility of detecting integration events and facilitate their incorporation into mechanistic and safety evaluations. However, in contrast to vector copy number (VCN), which has a defined regulatory cutoff, integration site analysis currently lacks standardized criteria for determining which insertion events are definitively oncogenic and should be excluded from infusion products. Even in the case of well-characterized oncogenes such as *TP53*, additional mutations are often required to drive malignant transformation, as demonstrated by Perica et al. (41). As such, integration site analysis is presently better suited for informational purposes rather than serving as a standalone lot-release assay.

Another critical genomic feature is the TCR repertoire, which has been increasingly recognized as a crucial factor influencing the treatment efficacy in immunotherapies (42–45). While CAR T-cell therapy primarily relies on the target CAR expression, endogenous TCR diversity, characterized by oligoclonality and polyclonality, within the infusion products may also contribute to treatment outcomes. Recent studies have characterized the kinetic profiling of different TCR clonotypes throughout the CAR T-cell treatment process (25, 46, 47), demonstrating the cytotoxic and proliferative features of highly expanded CAR T-cell clonotypes in patients. Paired single-cell RNA analysis and TCR repertoire profiling allow for the identification of individual CAR T-cells with distinct transcriptional phenotypes (48, 49), enabling the use of TCR clonotypes as surrogate for the expansion and persistence of functional T-cell states. Qing et al. applied this paired single-cell approach to 24 infusion products and found products associated with poor clinical responses exhibited moderately reduced TCR clonotypic diversity and showed exhaustion signatures (50).

γδ T-cells present another potentially advantageous subset in the infusion products. While the majority of CAR T-cell infusion products consist of αβ T-cells, γδ CAR T-cells have demonstrated resistance to exhaustion, exhibiting lower levels of *TIM3* and *PD1* expression following activation (51, 52). A longitudinal analysis of CD19 CAR T-cell therapy in a chronic lymphocytic leukemia patient who achieved a durable complete response revealed the expansion of a γδ CAR T-cell population, accounting for up to 33% of all CAR^+^ cells three months post-infusion (47). Our previous work also suggests that γδ T cell in CAR T-cell products may enhance cytotoxicity and be associated with favorable clinical responses (53). Collectively, these findings highlight the importance of TCR repertoire assessment in determining the potency of CAR T-cell infusion products.

### 2.2 Epigenomic profiles in CAR T-cell products

Preclinical and clinical trial data highlight the critical role of CAR T-cell differentiation states in determining therapeutic efficacy (54–56). CAR T-cell differentiation states refer to the developmental stages that T cells progress through, such as naïve, stem-cell like memory, central memory, effector memory, and terminally differentiated effector cells (57). These states are characterized by distinct gene expression profiles, functional properties, and persistence potential (58). T-cell differentiation is epigenetically programmed and maintained in progeny cells through chromatin states and DNA methylation (59). Insights from epigenomics studies have expanded our understanding of factors influencing CAR T-cell potential beyond transcriptomic profiling alone (60–70). Carlos et al. analyzed DNA methylation profiles in 114 CD19 CAR T-cell products and identified 18 distinct epigenetic loci associated with complete response (CR), event-free survival (EFS), and overall survival (OS) post-infusion. Using these CR-associated sites, they developed and validated an epigenetic signature, termed the EPICART signature, across different cohorts, demonstrating its potential as a predictor of CAR T-cell efficacy (62). Caitlin and colleagues performed a longitudinal DNA methylation assessment of CD8^+^ CD19 CAR T-cells from patients with B-cell acute lymphoblastic leukemia (B-ALL), revealing DNA methylation programs linked to a decline in CD19 CAR T-cell memory potential and the establishment of an exhaustion trajectory (63). These findings that have also been reported by others (64–66).

Epigenetic modulation has also been explored as a method to enhance CAR T-cell functionality. Yao et al. found CAR T-cells treated with low-dose decitabine (DAC, a *de novo* DNA methylation inhibitor) maintained higher memory-associated and lower exhaustion-associated gene expression profiles (64). Brooke and colleagues found that deleting *de novo* DNA methyltransferase 3 alpha (DNMT3A) in CAR T-cells prevented exhaustion and enhanced antitumor activity (65).

Beyond DNA methylation, histone modifications have been implicated in CAR T-cell function. Research has identified distinct histone markers that distinguish CD8^+^ T-cell subsets within CAR T-cell products (61). In preclinical investigations, Michel Sadelain’s group demonstrated that disrupting SUV39H1-mediated H3K9 methylation enhances the functional persistence of CD28-based CAR T-cells (67). Similarly, Mackall and colleagues restored functionality in exhausted CAR T-cells through epigenetic remodeling (68). Collectively, these findings underscore the importance of epigenomic profiling in understanding and optimizing CAR T-cell functionality. Beyond assessing product characteristics, epigenomic insights could inform potency assay development, guiding strategies to refine CAR T-cell manufacturing and enhance therapeutic efficacy. In the future, QC laboratories should consider implementing sequencing-based DNA methylation panels as potency assays, provided they can be robustly correlated with functional outputs. Alternatively, a single PCR-based assay targeting key epigenetic loci, such as those from the EPICART signature (62), could offer a more cost-effective and accessible option for routine potency testing.

### 2.3 Transcriptomics profiles in CAR T-cell products

Transcriptomics is a widely applied tool for analyzing gene expression (71–73), including in CAR T-cell products (5, 74–77). Both bulk and single-cell RNA sequencing (scRNA-seq) have emerged as powerful techniques for deciphering the molecular mechanisms governing CAR T-cell functionality, persistence, dysfunction, and therapeutic efficacy (50, 54, 78–82). To date, findings from transcriptomic profiling can be summarized into several key aspects.

#### 2.3.1 Distinct expression patterns correlate with clinical outcomes

Studies have reported that CD19 CAR T-cells from complete responders are enriched in memory-related gene signatures, including *IL-6/STAT3* signatures, whereas CAR T-cells from non-responders exhibit upregulated programs associated with effector differentiation, exhaustion and apoptosis (50, 82–84), which correlates with FACS data concerning CAR-T subsets. Additionally, CD19 CAR T-cells from CR patients demonstrate significantly higher expression of genes involved in glycolysis (82, 85). Preclinical studies further suggest that increased glycolytic activity in CAR T-cells is linked to enhanced potency, which may contribute to favorable efficacy (86, 87).

#### 2.3.2 Key transcriptional factors and regulons are associated with CAR T-cell function

Transcription factors (TFs) serve as master regulators of T-cell differentiation, expansion, fitness, and anti-tumor activity (88–90). Transcriptomic studies have identified several key TFs and associated regulons that play crucial roles in shaping CAR T-cell functionality (54, 91–96). One of the most well-characterized TFs in CAR T-cell biology is *TCF7* and its regulatory network, the *TCF7* regulon, which serves as a master regulator of T-cell memory. High *TCF7* expression has been linked to enhanced persistence and long-term efficacy in CAR T-cell therapy by maintaining a less-differentiated, stem-like phenotype associated with sustained antitumor activity (54, 93, 97). Another critical transcriptional network is *FOXO1* and its regulon (94, 95), which has been identified as a key enhancer of CAR T-cell function that boosts stemness, metabolic fitness, and antitumor activity. Additionally, *AP-1* family members play a significant role in modulating CAR T-cell exhaustion (98–100). *BATF* and *IRF4* cooperate to counter exhaustion in CAR T-cells (98), while *c-Jun* overexpression has been shown to induce resistance to exhaustion, thereby improving CAR T-cell functionality (99).

#### 2.3.3 Subsets of CAR T-cell populations are associated with clinical outcomes and long-term event-free survival

The advancement of single-cell RNA sequencing (scRNA-seq) has significantly enhanced our ability to uncovered previously unappreciated T-cell subsets in CAR T-cell infusion products, enabling the identification of minor yet functionally distinct CAR T-cell populations associated with clinical efficacy, highlighting their potential as biomarkers for potency assessment (22, 78, 81, 85). Less differentiated populations of CD8⁺ CAR T-cells, such as those with stem-like memory T-cell (Tscm) and central memory T-cell (Tcm) phenotypes, are associated with superior expansion, sustained tumor clearance, and prolonged EFS (50, 82, 101).

Emerging evidence suggests that a subset of cytotoxic CD4⁺ T cells in both infusion products and post-infusion samples, characterized by high expression of cytotoxic markers (*PRF1*, *GZMK*, *GZMB*, *NKG7*, and *GNLY*), correlates with clinical response (47, 50, 82, 102). Deng et al. found this subtype to be enriched in products with partial response (PR) and progressive disease (PD) (50). Maus’s team also reported CD4^+^NKG7^+^ cells were more prominent in non-responders (82). Moreover, Melenhorst and colleagues observed that in two patients who experienced decade-long remissions, cytotoxic CD4^+^ T-cells dominated the persistent population 5 to 10 years post-infusion (47). Regulatory T-cells (Tregs), expressing *FOXP3*, *IKZF2*, and *CTLA4*, have been observed in commercial CAR T-cell products and are suspected to contribute to CAR T-cell therapeutic resistance (82, 85, 102). Studies by Nicholas and colleagues and Good and colleagues found CAR-Treg cells were more frequent in non-responders and may contribute to relapse *in vivo (* 82, 102). Bai and colleagues studied CD19 CAR T-cell infusion products from 82 pediatric patients with B-ALL using scRNA-seq and CITE-seq. They found that Th2 function deficiency was associated with CD19-positive relapse, whereas Th2 functionality correlated with ultra-long-term event-free survival (EFS > 96 months) (79, 101, 103). A unique subset of CD8^+^ CAR T-cells termed CD8-fit T-cells, characterized by enhanced migration capacity, serial killing ability, and balanced mitochondrial and lysosomal volumes, has been identified (104). Infusion products with a higher proportion of CD8-fit T-cells correlated with favorable outcomes and long-term persistence in patients (104). Developing strategies to enrich CAR T-cell products with CD8-fit T-cells may significantly enhance clinical efficacy. Additionally, a double-negative T-cell phenotype was recently reported as a unique subset in infusion products that is associate with long-lived CAR T-cells (47, 78). Collectively, there is a need to develop manufacturing processes that select for desired CAR T-cell phenotypes and to establish potency assays that characterize gene expression patterns associated with positive clinical outcomes. These assays should be designed to provide actionable results in a timely manner, ensuring they effectively capture the unique characteristics of CAR T-cells and their therapeutic potential.

### 2.4 Proteomics profiles in CAR T-cell products

Advancements in proteomics profiling of CAR T-cell products have been driven primarily by mass cytometry, enabling the simultaneous characterization of intracellular signaling, activation, proliferation, cytokine production, and phenotype within a single assay (81, 105–111). Several studies have focused on deciphering CAR T-cell mechanisms of action using mass cytometry.

Goldberg and colleagues developed an integrative mass cytometry panel to analyze trafficking and functional protein expression in CD19 CAR T-cells (109), identifying upregulation of activation markers (*CD27*, *GZMB*, *CD69* and *CD25*), proliferation marker (*Ki-67*), and glycolysis markers (*Glut1*, and *LDHA*) in infusion products compared to baseline leukapheresis T-cells (81, 109). Salter and colleagues used mass spectrometry (MS)-based proteomics to reveal CAR T-cell activation pathways, including MAPKs (110). Hegde et al. using cytometry by time-of-flight (CyTOF) in HER-2 CAR T-cells, found lower frequencies of CD8^+^ T cells expressing *PD-1* ^+^ *TIM-3* ^+^, *PD-1* ^+^ *LAG-3* ^+^ or *PD-1* ^+^ *CD39* ^+^ in patients achieving CR, while higher and more variable levels were observed in those with SD and PD (108). Additionally, single-cell CyTOF analysis of day 7 circulating CAR T-cells in axi-cel-treated (a commercial CAR T-cell product) patients with large B-cell lymphoma identified three metaclusters associated with long-term clinical response (102).

### 2.5 Metabolomics profiles in CAR T-cell products

Unlike previous omics approaches, metabolomics focuses on cellular metabolism, including energy metabolism (glycolysis, oxidative phosphorylation, fatty acid oxidation, etc.), mitochondrial metabolism (biogenesis, fitness, ROS production, etc.), and the analysis of other metabolites. Recently, increasing attention has been given to metabolomics profiling of CAR T-cell products, which has provided crucial insights into how metabolic fitness influences persistence, cytotoxicity, and how this correlates with overall therapeutic efficacy (80, 86, 112–115). Oxidative phosphorylation (OXPHOS) is the predominant metabolic program in memory T-cells, while aerobic glycolysis characterizes effector T-cells (116, 117). Kawalekar and colleagues demonstrated that CAR T-cells with a CD28ζ costimulatory domain primarily rely on aerobic glycolysis, whereas those with BBζ preferentially utilize fatty acid oxidation (FAO), contributing to their enhanced persistence and central memory differentiation (118). Additionally, Cappabianca and colleagues found that metabolic priming by reducing aerobic glycolysis and increasing bound NAD(P)H activity was associated with lower cytokine production, including *IFN-γ*, *IL-2*, *IL-17*, and *TGF-β*, while promoting central memory CAR T-cell expansion and persistence in GD2 CAR T-cells (119).

As for the metabolites, Paul Renauer and colleagues identified *ADA* and *PDK1* as key metabolic regulators that enhance CAR19 T-cell cytolysis against leukemia cells (120). *ADA* (adenosine deaminase) catalyzes the conversion of adenosine to inosine within the purine metabolism pathway, and inosine has been reported to induce stemness features in CAR T-cells, enhancing their potency (121). Additionally, Ye and colleagues screened 27 differentially abundant metabolites in CD22 CAR T-cells with varying efficacy, identifying proline metabolism as the most significant contributor to CAR T-cell function (112). These findings suggest that detecting inosine or other metabolites in CAR T-cell supernatants could serve as a novel potency assay.

Mitochondrial properties have also been investigated in CAR T-cell products beyond energy metabolism. CAR T-cells from patients with a complete response exhibited increased mitochondrial biomass and volume compared to non-responders, correlating positively with expansion and persistence (104, 122). This enhanced persistence may be attributed to reserved bioenergetic potential, also known as spare respiratory capacity (SRC), which enables CAR T-cells to meet metabolic demand upon activation (112, 123). Additionally, mitochondrial quantities, ATP content, and the NADH/NAD ratio were found to be higher in Tscm, which are associated with superior efficacy compared to effector memory (Tem) and central memory (Tcm) T-cells (124). Overall, glycolysis, OXPHOS activity, metabolites in supernatant and mitochondrial indices could serve as potential markers for potency assay development.

### 2.6 Insights on developing potency assays

As CAR T-cell therapies advance, potency assays must evolve to accurately assess the complex characteristics of these cellular products. Insights from multi- omics approaches emphasize the need for more refined potency assays that effectively capture CAR T-cells efficacy and functionality (**Figure 2**). While further mechanistic studies are required to establish correlations between specific certain characteristics with a functional output, such as epigenomic profiles to IFN-γ secretion or cytotoxicity, it remains valuable to broadly explore CAR T-cell characteristics for their potential for potency assay development. Some of these characteristics require several days to complete which may prevent them from being used for potency assessment at this time. However, the field is progressing rapidly and if a specific profile is found to be useful for assessing CAR T-cell potency, alternative platforms that allow for more rapid testing will likely be developed. Here, we summarize key considerations for optimizing potency assays based on recent advancements.

#### 2.6.1 Adoption of customized panels in potency assay development

The implementation of customized panels for assessing key functional attributes, including gene expression (CAR T-cell stemness panel, CAR T-cell exhaustion panel, CAR T-cell persistence panel, etc.), DNA methylation [EPICART signature (62)], cytokine secretion (cytotoxic cytokines, Th2-skewed cytokines), surface marker expression (activation panel, exhaustion panel, stemness panel, etc.), and metabolite profiling, offers a promising approach for potency evaluation. Platforms like NanoString nCounter provide a rapid and robust solution for gene expression analysis using predefined panels. For cytokine detection, commercial panels from Bio-Rad, ProteinSimple, and MSD (Meso Scale Discovery) offer efficient and time-saving options. Additionally, surface marker detection has become more streamlined with high-throughput technologies such as flow cytometry, CyTOF, and Cytek platforms. Metabolite analysis can be performed using both targeted and untargeted panels available from providers like Metabolon Inc.

#### 2.6.2 Routine genomic CAR T-cell evaluation

NGS-based methods provide a powerful approach to evaluating CAR T-cells, enabling comprehensive analysis from vector integration sites to TCR repertoire, as well as from bulk and single-cell gene expression to chromatin accessibility. Integrating vector integration sites (Targeted DNA-seq) and TCR repertoire profiling (bulk TCR-seq and scTCR-seq) with gene expression data (RNA-seq, scRNA-seq, CITE-seq, etc.) allows for tracking clonal expansion and composition in CAR T-cells. Additionally, assessing histone modifications (ChIP-seq, CUT&Run-seq, etc.) and chromatin accessibility (ATAC-seq, DNase-seq, scATAC-seq, etc.) could be incorporated into QC pipelines to provide additional potency metrics. However, given the time-consuming nature of these methods, they may not be suitable for use as a “Lot-releasing assay”. However, the data could be used to engineer new manufacturing processes to enhance the desired characteristics identified by these assays.

#### 2.6.3 Routine monitoring of CAR T-cell metabolism

Metabolic fitness plays a critical role in CAR T-cell persistence and function, highlighting the need to integrate metabolic assessments into potency assays. Developing assays that evaluate glycolysis and OXPHOS activity in CAR T-cells, particularly before and after antigen stimulation *in vitro*, should be considered. The Seahorse XF Analyzer from Agilent provides a robust and efficient platform for real-time monitoring of these metabolic activities, offering insights into energy metabolism, mitochondrial function, and overall, CAR T-cell fitness. Routine metabolic monitoring could enhance CAR T-cell product characterization and contribute to guiding the development manufacturing of processes that produce products with the desired phenotypes.

## 3 Consideration in potency assays for unconventional CAR T-cells

**Figure 3:**
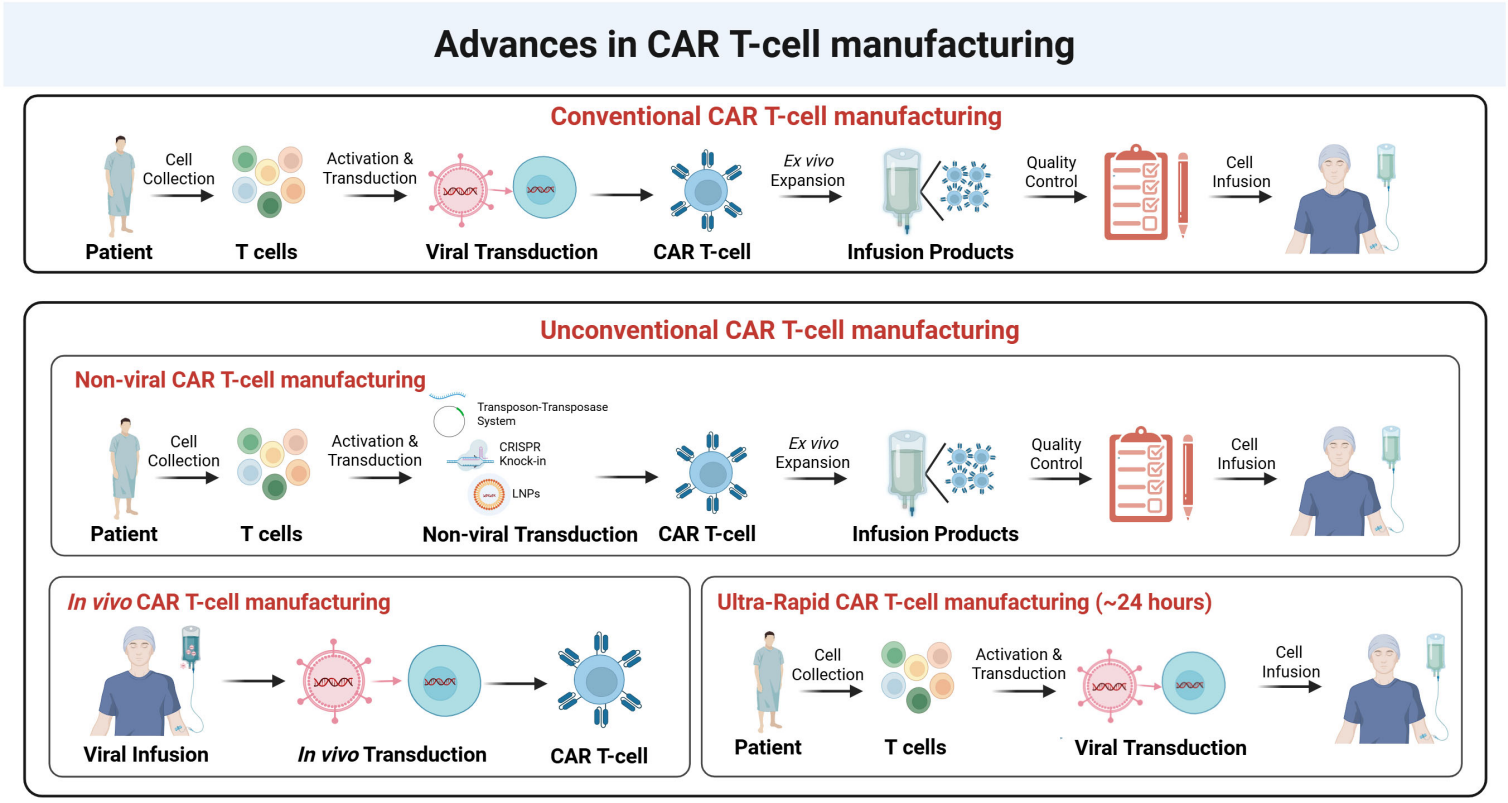
Advances in CAR T-cell manufacturing. Upper panel. Conventional CAR T-cell manufacturing involves the collection of T-cells from patients, activation, and viral transduction to introduce the CAR construct. The transduced CAR T-cells undergo ex vivo expansion, followed by quality control assessment before infusion into patients. Middle panel. Unconventional CAR T-cell manufacturing includes non-viral approaches, such as transposon-transposase systems, CRISPR knock-in, and lipid nanoparticle (LNP)-mediated delivery, as alternative strategies for CAR gene insertion. These non-viral methods follow a similar workflow of activation, transduction, expansion, and quality control before infusion. Bottom panel (left). In vivo CAR T-cell manufacturing eliminates the need for ex vivo manipulation by directly infusing viral vectors into the patient, allowing in vivo transduction and CAR T-cell generation within the body. Bottom panel (right). Ultra-rapid CAR T-cell manufacturing (~24 hours) aims to accelerate the process by minimizing ex vivo expansion steps, allowing rapid viral transduction and direct infusion into patients.

Real-world experiences with CAR T-cell therapies have highlighted the limitations of conventional manufacturing processes, which are often low-throughput, resource-intensive, and time-consuming (125–127). Traditionally, following apheresis, cells undergo activation, *ex vivo* modification, expansion, and rigorous quality control testing before infusion. Aiming to overcome these manufacturing challenges, in recent years, several unconventional manufacturing procedures have emerged, including non-viral CAR T-cell generation (128–131), *in vivo* CAR T-cell manufacturing (132–137), and rapid manufacturing protocols (138, 139). While these approaches offer advantages in efficiency, they also introduce new complexities for potency assays. In this section, we summarize recent advancements in CAR T-cell manufacturing and discuss key considerations for evaluating the potency of these unconventional CAR T-cells (**Figure 3**).

### 3.1 Non-viral CAR T-cells

The emergence of non-viral gene delivery methods (e.g., transposon systems, CRISPR-mediated knock-in) has provided alternative strategies for generating CAR T-cells without the need for viral vectors (140–142). These methods generally exhibit lower transduction efficiency than viral vector methods due to reduced knock-in rates, leading to a smaller proportion of CAR-expressing cells (140). In transposon-based systems (Sleeping beauty and PiggyBac), VCN varies widely due to uncontrolled transgene integration, resulting in heterogenous CAR expression across cells. Unlike viral vector-based CAR T-cells (VCN: 1–2 copies/cell), transposon-based CAR T-cells can exhibit 0-10+ copies/cell (18, 19, 130, 143, 144), raising concerns regarding product consistency and regulatory compliance. Given the FDA’s recommendation that the VCN should remain <5 copies/genome in infusion products (8), potency assays for transposon-based CAR T-cells must account for VCN thresholds to ensure safety and efficacy. For CRISPR-mediated CAR knock-in, off-target genome edits remain a significant concern, potentially affecting T-cell function and stability. Therefore, potency assays for these CAR T-cells should include whole-genome sequencing (WGS) (131, 145, 146) or GUIDE-seq (147, 148) to accurately identify and characterize these off-target sites.

### 3.2 *In-vivo* manufactured CAR T-cells

*In vivo* CAR T-cell generation eliminates the need for *ex vivo* expansion, shifting potency assessment from traditional pre-infusion characterization to real-time *in vivo* monitoring (149–151). In this instance, the viral vector itself is the primary product, making vector characterization an essential component of potency assay assessment. Conventional potency assays, such as those measuring transduction efficiency, cytotoxicity, and cytokine secretion *in vitro*, are no longer applicable. Future efforts *in vitro* potency analysis should focus on linking the characteristics of viral vectors and their functional outputs.

### 3.3 Ultra-rapid manufactured CAR T-cells

Ultra-rapid CAR T-cell manufacturing (3 days or less) (138, 152–154) significantly shortens the *ex vivo* expansion phase, introducing unique challenges for potency assessment. With some protocols completing the process in as little as 24 hours, the limited cell yield poses a challenge for conducting potency assays. Additionally, it remains uncertain whether these cells achieve sufficient CAR expression and vector copy number to be reliably detected by flow cytometry and ddPCR. Moreover, the shortened manufacturing time may result in a higher proportion of less-differentiated and less cytotoxic T-cells (153, 155), potentially biasing cytotoxicity potency assays for these CAR T-cells.

## 4 Summary

Ensuring the quality, consistency, and therapeutic efficacy of CAR T-cell products requires robust potency assays. Traditional potency assessments have been well-established for conventional CAR T-cell products. These assays focus on key parameters such as transduction efficiency, cytokine secretion, cytotoxicity. However, as CAR T-cell therapies continue to evolve, the emergence of new characteristics and manufacturing platforms necessitates a reassessment of current potency assays to ensure they remain accurate and relevant.

A critical aspect of potency assessment is the ability to accurately measure CAR T-cell functionality. While current *in vitro* assays provide valuable insights into cytotoxic activity and cytokine production, they may not fully capture the breadth of relevant cellular profiles. Additional profiles, such as vector integration events, T-cell differentiation state, and metabolic profiles, should also be considered when evaluating CAR T-cell potency. Advanced analytical techniques, including single-cell transcriptomics, high-dimensional flow cytometry, and metabolic analysis, offer more precise assessments of CAR T-cell function. These approaches help elucidate the complex interplay between CAR T-cell phenotype, functionality, and clinical outcomes, forming the foundation for developing robust potency assays.

The emergence of unconventional CAR T-cell manufacturing strategies, including non-viral gene delivery, *in vivo* CAR T-cell generation, and ultra-rapid manufacturing protocols, introduces new considerations for potency assessments. Non-viral CAR T-cells, while eliminating the need for viral vectors, exhibit greater variability in vector copy number and transgene integration, requiring refined potency assays to ensure product safety and efficacy. *In vivo* generated CAR T-cells shift potency evaluation from pre-infusion characterization to real-time *in vivo* monitoring, requiring novel biomarkers and functional assays to track their expansion and persistence post infusion. Ultra-rapid manufacturing, which significantly shortens *ex vivo* expansion time, poses challenges in achieving sufficient CAR expression and cell yield for traditional potency assays, necessitating innovative assay adaptations. Additionally, the development and usage of “off-the-shelf” allogeneic CAR T-cell products (129, 156, 157) introduces unique challenges related to donor suitability and ethical oversight. The application of genomic assays in this context may uncover clinically significant genomic lesions in donor cells, raising concerns about how to manage such findings in a way that ensures donor well-being while maintaining product quality. These evolving platforms highlight the need for a flexible and ethically informed potency assessment framework that can adapt to the changing landscape of CAR T-cell therapy.

Furthermore, the regulatory landscape for CAR T-cell potency testing continues to evolve. Regulatory agencies emphasize the importance of comprehensive characterization to ensure safety and efficacy while allowing for flexibility in adapting potency assays to emerging technologies. Establishing standardized potency criteria for diverse profiles and various CAR T-cell platforms will be essential for streamlining clinical translation and regulatory approval processes.

In addition to assessing the final CAR T-cell products, some omics methods can also be applied to the starting leukapheresis material (158). This upstream application is particularly valuable given the growing recognition that the functional fitness of the starting T-cell population strongly influences the quality and potency of the final CAR T-cell product. For example, profiling metabolic or protein expression signatures in pre-manufacture T cells may help predict manufacturing outcomes or therapeutic efficacy. Leveraging such assays early in the process could enable better donor or patient stratification, identification of optimal manufacturing candidates, and potentially guide pre-conditioning strategies to enhance T-cell fitness. Incorporating these omics approaches at the leukapheresis stage may therefore offer significant advantages for improving both the consistency and clinical performance of CAR T-cell therapies.

In conclusion, the rapid advancements in CAR T-cell therapy demand continuous refinement of potency assays to align with newly discovered characteristics and evolving manufacturing technologies. A multi-faceted approach that integrates traditional functional assays with cutting-edge analytical techniques will be crucial for accurately assessing CAR T-cell potency. By addressing these challenges, researchers and manufacturers can enhance the development of next-generation CAR T-cell therapies, ultimately improving patient outcomes in hematologic malignancies and solid tumors.

## Glossary

CAR T-cells: Chimeric Antigen Receptor T-cells
MoA: Mechanism of Action
FDA: Food and Drug Administration
NCI: National Cancer Institute
VCN: Vector Copy Number
ddPCR: Droplet Digital PCR
QC: Quality Control
CLL: Chronic Lymphoblastic Leukemia
ALL: Acute lymphoblastic Leukemia
LBCL: Large B-Cell Lymphoma
CR: Complete Response
PR: Partial Response
SD: Stable Disease
PD: Progressive Disease
EFS: Event-Free Survival
OS: Overall Survival
RNA-seq: RNA Sequencing
TCR-seq: T-cell Receptor Sequencing
scRNA-seq: Single-cell RNA-Sequencing
CITE-seq: Cellular Indexing of Transcriptomes and Epitopes Sequencing
ATAC-seq: Assays for Transposase-Accessible Chromatin with Sequencing
ChIP-seq: Chromatin Immunoprecipitation Sequencing
DNase-seq: DNase I Hypersensitive Sites Sequencing
CUT&Run-seq: Cleavage Under Targets and Release Using Nuclease Sequencing
Tn: Naïve T cells
Teff: Effector T cells
Tscm: Stem-like Memory T Cells
Tcm: Central Memory T Cells
Tem: Effector Memory T Cells
CyTOF: Cytometry by Time of Flight
MS: Mass Spectrometry
MC: Mass Cytometry
OXPHOS: Oxidative Phosphorylation
FAO: Fatty Acid Oxidation
SRC: Spare Respiratory Capacity
